# Chinese Herbal Complex ‘Bu Shen Jie Du Fang' (BSJDF) Modulated Autophagy in an MPP^+^-Induced Cell Model of Parkinson's Disease

**DOI:** 10.1155/2019/8920813

**Published:** 2019-03-13

**Authors:** Cuifang Liu, Xiaobo Huang, Shengxiang Qiu, Wenqiang Chen, Weihong Li, Haiyan Zhang, Tao Wang, Xue Wang, Xiling Wu

**Affiliations:** ^1^Department of Chinese Medicine, Xuanwu Hospital, Capital Medical University, Beijing, China; ^2^Key Laboratory of Plant Resources Conservation and Sustainable Utilization, Guangdong Provincial Key Laboratory of Applied Botany, South China Botanical Garden, Chinese Academy of Sciences, Guangzhou, China; ^3^Department of Cell Biology, Capital Medical University, Beijing, China; ^4^Department of Neurosurgery, Xuanwu Hospital, Capital Medical University, Beijing, China; ^5^Department of library, Xuanwu Hospital, Capital Medical University, Beijing, China

## Abstract

Autophagy plays an important role in the development of Parkinson disease (PD). Previous studies showed that autophagy could protect cells from *α*-synuclein toxicity and promote functional coupling of mitochondria. But it is still a question whether modulating autophagy can be used to treat PD. In traditional Chinese medicine, a specific Chinese herbal complex called Bu Shen Jie Du Fang (BSJDF) has a long history of treating motor impairments similar to Parkinson disease, while its mechanism is still unclear. As a pilot study, we aimed to evaluate the efficacy and its mechanism of Bu Shen Jie Du Fang in an MPP^+^-induced cell model of Parkinson's disease. And the phase contrast microscope (PCM) revealed that the BSJDF group had the greatest surviving cell counts compared with all other treated cell groups except the normal group. And Cell Counting Kit 8 (CCK8) assays showed a similar result. In BSJDF group, 3.7 ×10^7^ cells/dish was identified by hemocytometer counts, which was significantly higher than other groups except the normal cells (p<0.05). In the BSJDF group, autophagy can be observed by transmission electron microscopy (TEM). Protein expression of Atg12 and LC3 in the BSJDF group was upregulated compared to the PD model group (p<0.05). Atg12 mRNA expression was also upregulated in the BSJDF group (p<0.05). In conclusion, our study indicated that the therapeutic mechanisms of BSJDF may be mediated by stimulating autophagy, and modulating autophagy can be used to treat PD.

## 1. Introduction

Parkinson's disease (PD) is a progressive, degenerative neurologic disease characterized by a tremor that is maximal at rest, slowness of movement, rigidity, and postural instability [1]. Its incidence is 8 to 18 per 100,000 person-years based on prospective population-based studies with either record-based or in-person case finding [2–4]. Currently, the therapy is limited, only capable of slowing down the progression of this disabling neurodegenerative disorder [5]. Drug therapy and surgery mainly target impairments related to dopaminergic lesions [6]. L-DOPA, used as the pharmacological replacement of dopamine (DA), is the major pharmaceutical drug for symptom control, but most patients still experience motor fluctuations as the disease progresses [5, 7]. Moreover, drug therapy becomes less effective or causes complications like disabling dyskinesias in later stages of the disease [6].

The etiology and pathology of the disease remain largely unknown. Many studies have addressed the issue of autophagy in PD. Previous studies suggested that dysregulation of autophagy is implicated in the pathogenesis of idiopathic and familial PD [8, 9]. Ravikumar and colleagues [10] demonstrated* in vivo* that enhancing autophagy provided protection in neurodegeneration. Whether “excessive” autophagy mediates “autophagic cell death” is perhaps one of the most controversial areas in autophagy research [11]. Autophagy plays a complex role in PD, and it may play a fundamental role in the development of disease, thus its regulation may be important to find a new therapy [8, 11]. In the autophagy signaling, Beclin-1, LC3, and Atg12 play essential roles in the initiation step of autophagy. Beclin-1 serves as a critical regulator at the beginning of autophagic vesicle nucleation [12]. Beclin1-PI3KC3 is an essential complex for autophagosome formation, which plays a role in nucleation and initial phagophore membrane formation [13]. LC3 (the mammalian homolog of Atg8), which have two cellular forms, LC3-I (a cytoplasmic form of LC3) and LC3-II (a cleaved form) [14], was considered as a specific marker for autophagy [14]. Except the Atg8/LC3 lipidation system, the Atg5-Atg12 conjugation system also plays essential roles in autophagosome initiation and expansion [13]. Atg12 is also involved in autophagosome formation [15]. So Beclin-1, LC3, and Atg12 are useful representative proteins for investigating autophagy.

Traditional Chinese medicine, including a specific Chinese herbal complex called Bu Shen Jie Du Fang (BSJDF), has a long history of treating motor impairments similar to Parkinson disease. Clinical observations showed that BSJDF enhances functional capacity in PD patients, without leading to motor fluctuations. Bu Shen Jie Du Fang (BSJDF) is composed of* Rehmannia glutinosa*,* Cistanche deserticola*,* Paeonia lactiflora Pall*,* Radix Angelica sinensis*,* Puerariae Radix*,* Rhizoma Coptidis, Radix Scutellariae*,* Antelope Horn Powder*, and* Glycyrrhizae Radix *in a weight ratio of 5:5:4:4:5:4:4:1:2. Earlier reports revealed that* Radix Scutellariae* induced autophagic cell death in SMMC-7721 cells [16, 17];* Rhizoma Coptidis *stimulated autophagy and suppressed the proinflammatory phenotype of macrophages [18]. Furthermore,* Glycyrrhizae Radix* has been shown to induce autophagic cell death in cervical and breast cancer, as well as androgen-sensitive prostate adenocarcinomas and adenoid cystic carcinoma cancer cells [19]. The present study was designed to explore the therapeutic mechanisms of BSJDF: whether it is mediated by stimulating autophagy and whether stimulating autophagy can be used to treat PD. We investigated the effect of BSJDF on pheochromocytoma 12 (PC12) cells treated with the neurotoxin MPP^+^ (a metabolite of MPTP (1-methyl-4-phenyl-1,2,5,6tetrahydropyridine) to induce PD* in vivo *[3, 20–22].

## 2. Results

### 2.1. Cell Survival Was Increased in the BSJDF Group

**Figure 1:**
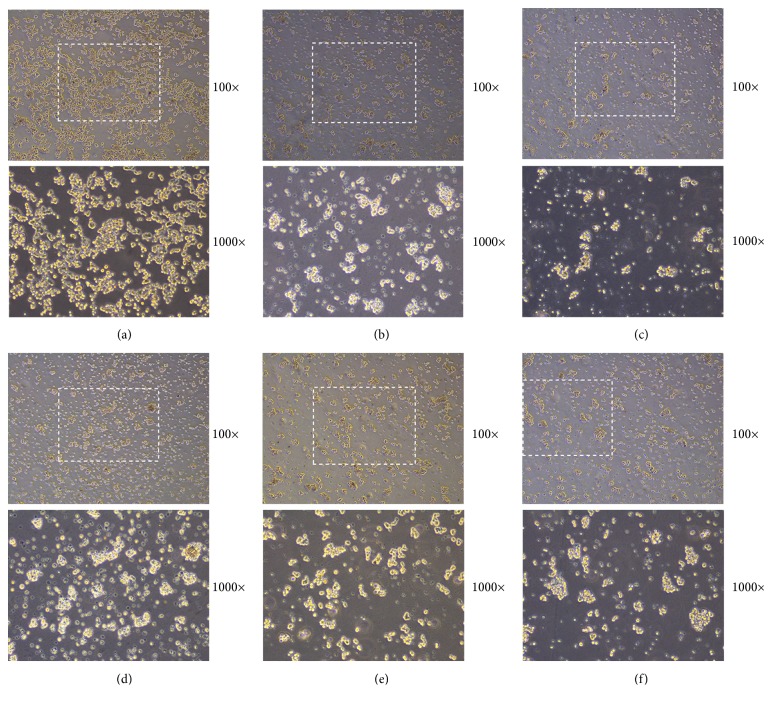
The cells were reviewed under PCM enlarged 100× and 1000×. (a) The normal group which is untreated PC12 cells; (b) the rapamycin group treated with MPP^+^ for 24 h and then rapamycin for 24 h; (c) the PD model group treated with MPP^+^ for 48 h; (d) the BSJDF group treated with MPP^+^ for 24 h and then BSJDF serum for 24 h; (e) the 3-MA group treated with MPP^+^ for 24 h and then 3-MA for 24 h; (f) the NH_4_CL group treated with MPP^+^ for 24 h and then NH_4_Cl for 24 h. The cell number in the BSJDF group is larger than the others except the normal group.

**Figure 2:**
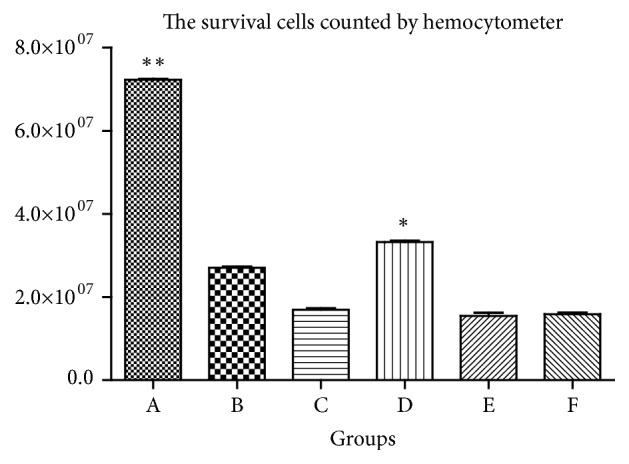
The survival cells counted by hemocytometer. A, the normal group, the survival cells were 7.3 × 10^7^; B, the rapamycin group, the survival cells were 2.8 × 10^7^; C, the PD model group, the survival cells were 1.8 × 10^7^; D, the BSJDF group, the survival cells were 3.4 × 10^7^; E, the 3-MA group, the survival cells were 1.6 × 10^7^; F, the NH_4_CL group, the survival cells were 1.6 × 10^7^; the number of cells surviving in the BSJDF group was larger than other groups except the normal group (p<0.05), *∗*p<0.05 and *∗∗*p<0.01.

**Figure 3:**
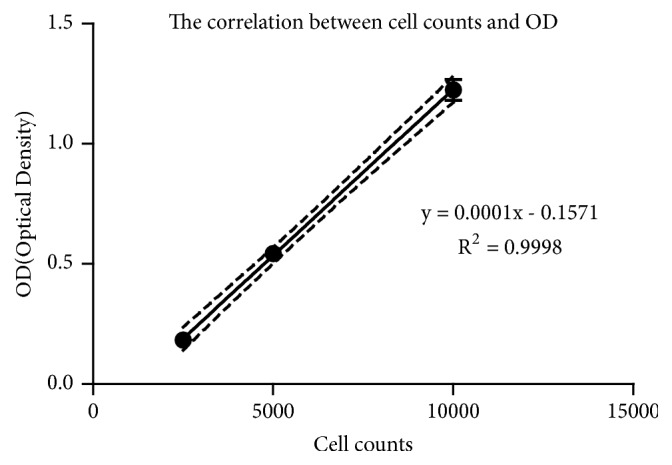
The correlation between the counts and OD (optical density) (R^2^ = 0.9998).

**Figure 4:**
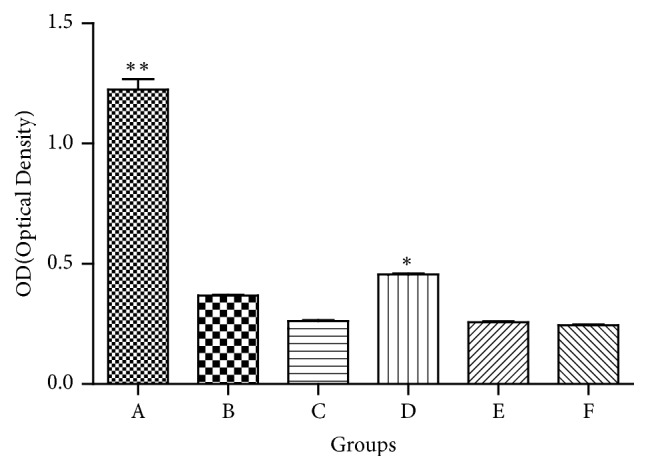
The OD results in different groups by CCK8. A, the normal group; B, the rapamycin group; C, the PD model group; D, the BSJDF group; E, the 3-MA group; F, the NH_4_CL group. The number of cells surviving in the BSJDF group has more cell counts than the others, except the normal group (p<0.05), *∗*p<0.05 and *∗∗*p<0.01.

We assessed the effect of cotreating PC12 cells with different drugs by cell survival counts at 48 h. The PCM (phase contrast microscope) demonstrated that the highest number of survival cells in the BSJDF group rather than the others, except the normal group (Figure 1(d)). We counted the survival cells by hemocytometer and then calculated and analyzed the results. And the statistics also showed that BSJDF group has the maximum surviving cells compared with other groups except the normal group (Figure 2 D). Cell Counting Kit 8 (CCK-8) revealed the correlation between the counts and OD (Optical Density, which is absorbance values) (Figure 3). The result proved that BSJDF group had the largest OD than all other groups except the normal group (Figure 4 D). And BSJDF group had the greatest surviving cell counts compared with all other groups except the normal group.

### 2.2. The Autophagy Was Clearly Observed by Transmission Electron Microscopy (TEM) in Rapamycin Group and BSJDF Group

**Figure 5:**
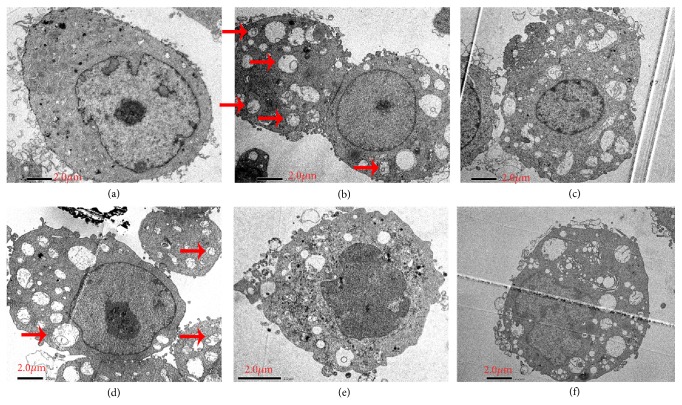
The formation of autophagosomes in treated cells was evaluated by TEM. (a) the normal group; (b) the rapamycin group; (c) the PD model group; (d) the BSJDF group; (e) the 3-MA group; (f) the NH_4_CL group. The arrowheads autophagy (scale bar: 2.0 *μ*m). “Red arrow” indicates autophagosomes.

To investigate whether BSJDF induces autophagy, PC12 cells were assigned to groups and treated for 48 h, as described above, before TEM examination. As we know, autophagy was indicated as double-membrane enclosed autophagosomes by TEM, and we can observe autophagy in the rapamycin group (Figure 5(b); red arrow) and the BSJDF group (Figure 5(d); red arrow). TEM result also showed the cells in PD model group and 3-MA group exhibited impaired autophagy (Figures 5(c) and 5(e)). NH_4_Cl markedly increased the number of these intracellular vacuoles (Figure 5(f))

### 2.3. Protein Expression of Atg12 and LC3 Was Increased by BSJDF

**Figure 6:**
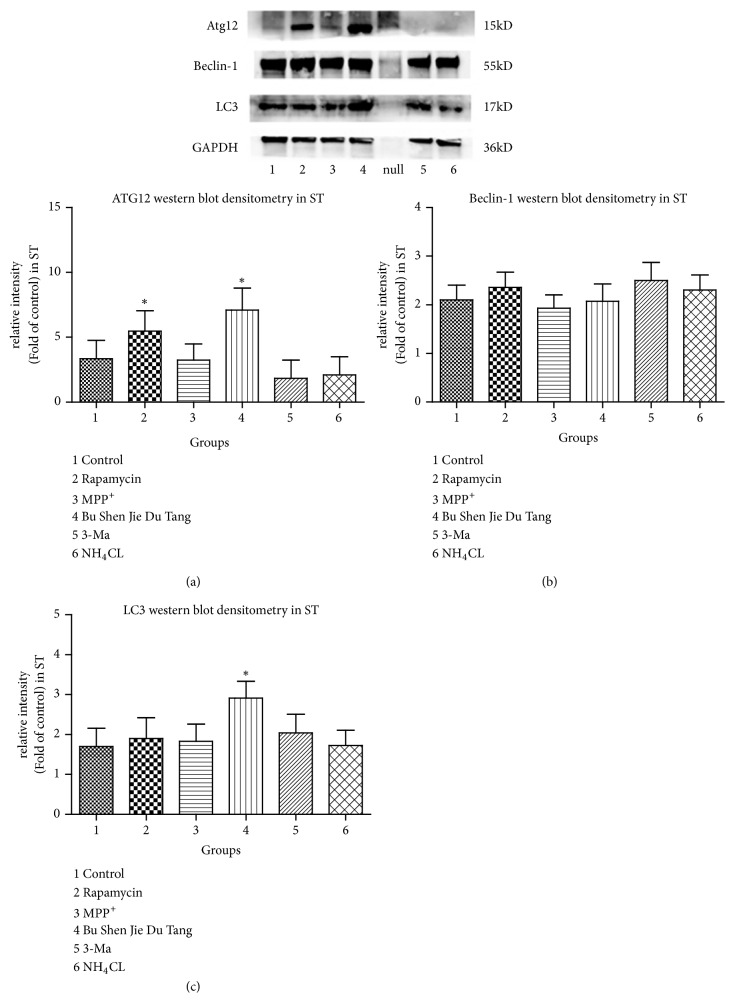
1, the normal group; 2, the rapamycin group; 3, the PD model group; 4, the BSJDF group; 5, the 3-MA group; 6, the NH_4_CL group. GAPDH was used as a loading control. (a) Atg12 western blot densitometry in ST. (b) Beclin-1 western blot densitometry in ST. (c) LC3 western blot densitometry in ST. *∗*p<0.05 and *∗∗*p<0.01.

To investigate whether cell survival following BSJDF treatment was mediated by autophagy, we analyzed Beclin-1, Atg12, and LC3 expression by western blotting. And the result showed that protein expression of Atg12 and LC3 was upregulated in the BSJDF group. The remarkable increased Atg12 expression had a statistically significant difference (p<0.05) compared with other groups except the rapamycin group (Figure 6(a)). The expression of Atg12 between rapamycin group and BSJDF group did not show a statistically significant difference (p>0.05). The upregulated LC3 expression also had a statistically significant difference (p<0.05) (Figure 6(c)). Also, we can find that Atg12 expression was significantly higher (p<0.05) in rapamycin group except BSJDF group (Figure 6(a)).

### 2.4. mRNA Expression of LC3 Was Upregulated by BSJDF

**Figure 7:**
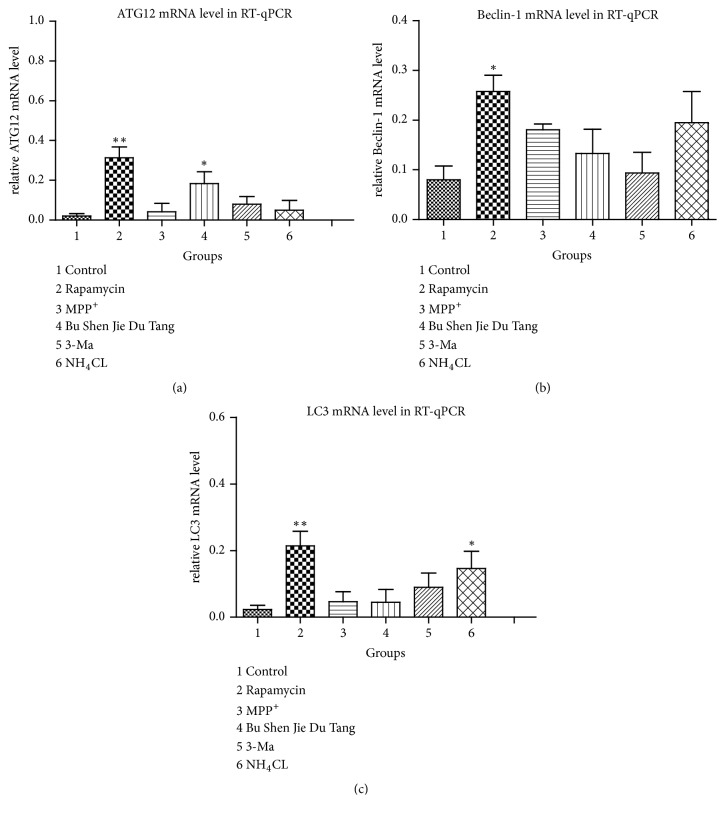
1, the normal group; 2, the rapamycin group; 3, the PD model group; 4, the BSJDF group; 5, the 3-MA group; 6, the NH_4_CL group. GAPDH was used as a loading control. (a) Atg12 mRNA expression level in RT-qPCR. (b) Beclin-1 mRNA expression level in RT-qPCR. (c) LC3 mRNA expression level in RT-qPCR. *∗*p<0.05 and *∗∗*p<0.01.

RT-qPCR revealed a significantly higher mRNA expression of Atg12 in the BSJDF group than other groups except the rapamycin group (p<0.05) (Figure 7(a)). In the rapamycin group, we observed a higher mRNA expression of Atg12, which had a statistically significant difference (p<0.01) compared with other groups, except the BSJDF group (Figure 7(a)). Not only the Atg12 mRNA but also Beclin-1 showed higher expression which had a statistically significant difference (p<0.05), as did LC3 mRNA expression (p<0.01), in the rapamycin group compared with the others (Figure 7(c)).

## 3. Discussion

In the present study, we explored the underlying modulated mechanism of BSJDF on the principal aspect autophagy relating mRNA and proteins. We examined the neuroprotective effects of BSJDF by PCM, hemocytometer, and CCK-8. And autophagic process was visualized using TEM. To elucidate the possible mechanisms of how BSJDF regulates autophagy, we performed western blotting and RT-qPCR. Our results showed that BSJDF can protect PC12 cells (Figures 1, 2, and 4 D) which was based a higher cervical cells counts than other groups except the normal condition in BSJDF group by PCM, hemocytometer, and CCK-8. And BSJDF could activate autophagy (Figure 5(d)) by TEM. BSJDF also increased protein expression of Atg12 and LC3 (Figures 6(a) and 6(c)) and upregulated Atg12 mRNA expression (Figure 7(a)). Collectively, these findings indicate that BSJDF improves cell survival by inducing autophagy in the PC12 cell PD model. Autophagy plays an important role in cell fate and maintaining cellular metabolic balance [9]. Anglade et al. (1997) identified apoptosis and autophagy as a possible mechanism when they discovered that nigrostriatal neurons were lost in PD patients [23]. Since then, many studies have reported that autophagy exerted an important protective effect on neurons [15, 24, 25]. Autophagy-mediated clearance of aggresomes has been implicated in many neurodegenerative diseases, and autophagy regulation is considered as a potential method in treatment of PD [26–29]. Indeed, autophagy is a key step in the development of PD. In our research, we established an* in vitro* PD model in MPP^+^-induced PC12 cells, which provided a stable and reliable assay for estimating the effects of anti-PD drugs [28].

We did not observe an increase in the protein or mRNA expression of Beclin-1 (Figures 6(b) and 7(b)), Beclin-1 overexpression via lentivirus delivery is beneficial for PD [30] in the setting of BSJDF treatment. But BSJDF can increase Atg12 and LC3 protein expression (Figures 6(a) and 6(c)) and upregulate Atg12 mRNA expression (Figure 7(a)). Interestingly, Atg12 protein overexpression inhibits autophagosome formation in HEK-293 cells [31], and changing Atg12 protein levels contributes to the development of sporadic PD [15]. Meanwhile, upregulation of LC3 protein is sufficient to enhance autophagic activity and reduce the accumulation of *α*‐synuclein (SNCA, a key player in PD)* in vitro* and* in vivo *[28]. So, based on the increased protein expression of Atg12 and LC3 and upregulated mRNA expression of Atg12, we hypothesize that BSJDF improve cell survival in the PC12 model of PD by inducing autophagy.

Upon comparing the PCM, hemocytometer, CCK-8, and TEM results, we found the rapamycin-treated cells that showed autophagy under TEM (Figure 5(b)), which had fewer survival cells compared to the BSJDF group (Figures 1, 2, and 4 B). We therefore hypothesize that in addition to inducing autophagy (increased expression of Atg12 and LC3 protein), BSJDF has other effects such as regulating the balance of autophagy to provide protection in a cell model of PD, which can be further confirmed in clinical trials. Further research is needed to determine the key material in BSJDF which is responsible for its neuroprotective effect.

## 4. Materials and Methods

### 4.1. The Preparation of Materials and BSJDF Serum

#### 4.1.1. Medications

The component substances used to make BSJDF were as follows:* Rehmannia glutinosa*,* Cistanche deserticola*,* Paeonia lactiflora Pall*,* Radix Angelica Sinensis*,* Puerariae Radix*,* Coptidis Rhizoma*,* Scutellariae Radix*,* Antelope Horn Powder*, and* Glycyrrhiza uralensis*. These herbs were mixed based on a dry weight ratio of 5:5:4:4:5:4:4:1:2, respectively. All of the plants were extracted by standard methods according to the Chinese Pharmacopoeia. The manufacturing process of BSJDF begins with decoction. Next, the filtrate is sent for composition analysis by HPLC.

All herbs were purchased from the Pharmacy Department of Xuanwu Hospital, Beijing, China, and were authenticated by doctor Xiling Wu, Department of Traditional Chinese medicine, Xuanwu Hospital of Capital Medical University, China (Table 1). The authenticated voucher specimens are available in the Pharmacy Department of Xuanwu Hospital.

The ingredients (except for Antelope horn powder) were immersed in 10-fold volumes of water for 30 min and decocted twice in the same solution at 100°C for 30 min. The decocted solutions were mixed together, and the Antelope horn powder was added to produce BSJDF, which was stored in the refrigerator for later use at 4°C. The final concentration of the solution was 0.255 g/mL (equivalent to the dry weight of raw materials in 400 ml liquid).

#### 4.1.2. BSJDF Serum Preparation

Ten Sprague–Dawley rats (5 males, 5 females) were purchased from the Laboratory Animal Center of Xuanwu Hospital, Beijing, China. All animal experiment were approved by the Institutional Animal Care and Use Committee of Xuanwu Hospital, Capital Medical University, China, and conducted according to guidelines laid out by the National Institutes of Health. Animals initially weighed 200-250 g and were housed individually at 21°C with a 12 h:12 h light/dark cycle. Rats had free access to standard food and drinking water.

Animals were treated with BSJDF (5.1 g/kg body weight per day) for 3 d, by oral administration. Blood was collected from the abdominal aorta 2 h after the final administration and then centrifuged at 3000 rpm for 20 min to obtain serum. The serum was heated in a 56°C water bath for 30 min and then stored at −20°C before further analysis [32].

#### 4.1.3. Reagents and Antibodies

Cell culture medium (Dulbecco's minimum essential medium, DMEM) RIMP1640, fetal bovine serum (FBS), heat-inactivated horse serum, and penicillin-streptomycin liquid were obtained from Gibco (Grand Island, NY, USA). Rabbit anti-Beclin-1 antibody, rabbit anti-Atg12 antibody, rabbit anti-LC3A/B antibody, rabbit anti-GAPDH antibody, and goat anti-rabbit IgG H&L were obtained from Abcam (Cambridge, UK). We purchased MPP+ iodide and NH_4_CL from Sigma Co. (St. Louis, MO, USA). All other materials were purchased from Sigma Co., except where indicated, and were of analytical grade. ExpressPlus PAGE Gels and Tris-MOPS-SDS Running Buffer Powder were purchased from GenScript (Nanjing, China). The RNAprep pure cell kit, FastKing RT Kit (with gDNase), and Talent qPCR PreMix (SYBR Green) were purchased from TIANGEN (Beijing, China)

### 4.2. Cell Culture and Cell Counts

Rat PC12 cells (Cat. 3111C0001CCC000024; National Infrastructure of Cell Line Resource, China) were cultured in DMEM supplemented with 5% FBS and 10% heat-inactivated horse serum, before adding 100 *μ*g/mL penicillin-streptomycin liquid to the medium. The cells were cultured at 37°C in a humidified mix of 5% CO_2_ and 95% air. Cells were split 1:4 every 3 d; experiments were performed on cells at passages 4 to 8 when they were at 80-90% confluence. All experiments were carried out 24-48 h after the cells were seeded [33].

We divided cells into six treatment groups: (1) the normal group were cultured with 5% FBS and 10% heat-inactivated horse serum; (2) the MPP^+^ PD model control group were treated with 200 mM MPP^+^ in 5% FBS and 10% heat-inactivated horse serum for 48 h; (3) the rapamycin group, which can induce autophagy in a variety of cell types [34] via its methoxy group, were treated with 200 mM MPP^+^ in 5% FBS and 10% heat-inactivated horse serum for 24 h, before being cocultured with 100 nM rapamycin (Cell Signaling Technology, Beverly, MA, USA) for 24 h; (4) the BSJDF group were treated with 200 mM MPP^+^ in 5% FBS and 10% heat-inactivated horse serum for 24 h, before being cocultured with 10% BSJDF serum for 24 h; (5) the 3-methyladenine (3-MA) group, which as a PI3K and autophagy inhibitor [35] is the most commonly used specific inhibitor of autophagic removal[36], were treated with 200 mM MPP^+^ in 5% FBS and 10% heat-inactivated horse serum for 24 h and then cocultured with 15 mM 3-MA (Sigma) for 24 h; and (6) the NH_4_Cl (ammonium chloride) group, a specific and rapid inhibitor of induced autophagy-mediated proteolysis and inhibits autolysosome formation [37], were treated with 200 mM MPP^+^ in 5% FBS and 10% heat-inactivated horse serum for 24 h and then cocultured with 10 mM NH_4_Cl (Cell Signaling Technology) for 24 h. After 48 h, all cells were examined using a phase contrast microscope (PCM; Axio Observer 3m; Carl Zeiss, Jena, Germany) under 100× and 1000× magnification and the survival cells counted by hemocytometer.

Cell counts were assessed by Cell Counting Kit 8 (CCK-8; Solarbio, China) assays according to the manufacturer's instructions. First, we divided PC12 cells into three groups and labeled. In the first group we placed six wells of 96-well plate at a density of 1 × 10^4^ peer plate, in the second group 5 × 10^3^ cells per well were plated into 96-well plate in six wells, and in the third group 2.5 × 10^2^ cells per well were plated into 96-well plate in six wells. Cells were cultured in complete medium (DMEM including10% fetal bovine serum (FBS) and 5% heat-inactivated horse serum) at 37°C in 5% CO_2_. 10 *μ*L CCK8 was added for 2 h. The spectrometric absorbance was measured by microplate reader (Thermo Scientific, Massachusetts, US) at 450 nm and then analyzed the correlation between the counts and absorbance values and marked a standard curve.

PC12 cells of six difference treatment groups were plated into 96-well plate with 1 × 10^4^ cells per well in six wells and cultured in corresponding medium at 37°C in 5% CO_2_. Then 10 *μ*L CCK8 solution was added for 24 h and absorbance values were determined at 450 nm. and spectrometric absorbance was measured by using microplate reader at a wavelength of 450 nm. Depending on the standard curve, the system calculates the number of different groups in the sample.

### 4.3. Observing Autophagy by Transmission Electron Microscopy (TEM)

Autophagosome morphology was examined with TEM. After 48 h, PC12 cells were fixed in ice-cold 2.5% glutaraldehyde in 0.1 mol/L phosphate-buffered saline (PBS) at 4°C for 1.5-2 h. Then cells were postfixed for 1 h in 1% osmium tetroxide in the same buffer, dehydrated in graded alcohols and acetones, and embedded in Epon 812 at the laboratory of Capital Medical University (Beijing, China). Samples were sectioned with an LKB-I ultramicrotome in 50 to 60 nm thick slices. Then the sections were stained with 3% uranyl acetate and lead citrate and examined with a transmission electron microscope (JEM-1400plus, JEOL, Tokyo, Japan).

### 4.4. Protein and mRNA Expression

We measured the protein and mRNA expression of Beclin-1, Atg12, LC3. Protein was extracted as follows: cell culture solutions were centrifuged at 800 rpm for 5 min, the supernatant was discarded, cells were collected, radioimmunoprecipitation assay lysis buffer (Beyotime, Shanghai, China) in phenylmethanesulfonyl fluoride was added, and the resultant solutions were placed on ice for 30 min. The supernatants were collected after centrifugation at 12,000* g *at 4°C for 20 min. Protein concentration was determined using a BCA Protein Assay Kit (Beyotime, Shanghai, China), and whole lysates were mixed with 5× loading buffer at a ratio of 1:5. Samples were heated at 95°C for 15 min and were separated on SDS–polyacrylamide gels. The separated proteins were then transferred to polyvinylidene difluoride membranes. The blots were first probed with a primary antibody. After incubation with horseradish peroxidase- (HRP-) conjugated secondary antibody, autoradiograms were prepared using an enhanced chemiluminescent system to visualize the protein antigen. The signals were recorded using X-ray film. The primary antibodies were rabbit anti-LC3, anti-Beclin-1, anti-ATG12, and antiglyceraldehyde 3-phosphate dehydrogenase (GAPDH). The secondary antibody was goat anti-rabbit. We used GAPDH as a protein loading control. Protein levels were first normalized to GAPDH and then normalized to the experimental control. Images shown in the figures represent data from five animals. Western blot densitometry was performed with AlphaView software.

Total RNA was extracted from PC12 cells using the RNAprep pure cell kit; then the RNA was reverse transcribed to cDNA using the FastKing RT Kit (with gDNase). We conducted cDNA synthesis in a reaction mixture containing 2 *μ*l 5× gDNA buffer, 2 *μ*l total RNA, and 6 *μ*l RNase-free ddH_2_O. The total reaction volume of 10 ml was mixed, centrifuged for 30 s, and stained at 42°C for 3 min. Then we added another reaction mixture containing 2 *μ*l 10× King RT Buffer, 2 *μ*l FastKing RT Enzyme Mix, 2 *μ*l FQ-RT Primer Mix, and 5 *μ*l RNase-free ddH_2_O. The total reaction volume was 20 ml. Reverse transcription was performed at 42°C for 30 min and then 99°C for 5 min and stored at 20°C for RT-qPCR. The details of all oligonucleotide primer sequences, predicted product lengths, and GenBank accession numbers for sequences amplified by RT-PCR are listed in Table 2. The RT-PCR reaction mixture using the Talent qPCR PreMix (SYBR Green) consisted of 10 *μ*l 2× Talent qPCR Premix, 0.6 *μ*l forward primer, 0.6 *μ*l reverse primer, 1 *μ*l cDNA, and 7.8 *μ*l RNase-free ddH_2_O. The total reaction volume was 20 ml. The reaction was performed under the following conditions: predenaturation at 95°C for 5 min, denaturation at 95°C for 10 s, annealing at 60°C for 10 s, extension at 72°C for 20 s for a total of 45 cycles and then denaturation at 95°C for 5 s, annealing at 65°C for 1 min, denaturation at 97°C, continuous for a total of 1 cycles; then a final extension step was performed at 40°C for 30 s. An RT-PCR assay was carried out with a Roche LightCycler480 (Basel, Switzerland).

### 4.5. Statistical Analysis

All statistical analyses were carried out using GraphPad Prism (GraphPad Software, La Jolla, CA, USA). Data were analyzed using SPSS (SPSS 22.0 for Windows, IBM Corp., Armonk, NY) and expressed as mean ± SD of the indicated number of independent experiments. Statistical significance among groups was evaluated by a one-way analysis of variance (ANOVA) followed by Bonferroni-corrected comparison tests. A value of* p *< 0.05 was considered statistically significant for all tests.

## 5. Conclusions

In conclusion, this study is the first to investigate the possible mechanism of autophagy signaling, through which BSJDF improved survival in the PC12 cell model of PD. Our findings indicated that BSJDF improves MPP^+^-induced injury. We found that BSJDF protected PC12 cells by inducing autophagy. However, its effect was not solely attributed to autophagy induction because a greater number of cells survived following treatment with BSJDF than rapamycin, an autophagy inducer (Figures 1, 2, and 4 B, D). We therefore hypothesize that BSJDF regulates the balance of autophagy, but the specific underlying mechanism remains to be elucidated. Our research provides a new way, which is worth going into additional research, for the development of PD medicine in the future.

## Figures and Tables

**Table 1:** Component herbs of BSJDF.

	Botanical plant name	family	Part used	Ratio of composition
*Rehmannia glutinosa*	Rehmannia glutinosa(Gdertn)	Scrophulariaceae	root and rhizome	5
*Cistanche deserticola*	Cistanche deserticola Y.C.Ma	Orobanchaceae	Fleshy stem	5
*Paeonia lactiflora Pall*	Raeonia lactiflora pall	Paeoniaceae	Radix	4
*Radix Angelic Sinensis*	Aaugellica sinensis(Oliv) Diels	Apiaceae	Radix	4
*Puerariae Radix*	Pueraria lobata	Fabaceae	Radix	5
*Coptidis Rhizoma*	Coptis chinensis Franch	Ranunculaceae	Rhizome	4
*Scutellariae Radix*	Scutellaria baicalensis Georgi	Labiatae	Radix	4
*Cornu Bubali*	Cornu Bubali	Cornu Bubali	Horn	1
*Glycyrrhizae radix*	Glycyrrhiza uralensis	Leguminosae	Radix	2

**Table 2:** Sequences of primers used for RT-PCR.

Name	Oligo	Primer sequence	Predicted size(bp)	Genebank accession
Atg12	Forward primer	aaacgaagaaatgggctgtg	148	
	Reverse primer	gtcccaacttcttggtctgg	148	NM_001038495.1
LC3	Forward primer	gcctgtcctggataagacca	121	
	Reverse primer	gttcaccagcaggaagaagg	121	NM_012823.2
Beclin-1	Forward primer	ggccaataagatgggtctga	182	
	Reverse primer	gctgcacacagtccagaaaa	182	NM_001034117.1
*β*-actin	Forward primer	gctgacaggatgcagaagga	124	
	Reverse primer	tggacagtgaggccaggata	124	NM_031144
GAPDH	Forward primer	cctgcaccaccaactgctta	120	
	Reverse primer	ggccatccacagtcttctga	120	NM_017008

## Data Availability

The data used to support the findings of this study are included within the article.
